# Atherosclerosis in Juvenile Idiopathic Arthritis

**DOI:** 10.1155/2012/714732

**Published:** 2012-08-13

**Authors:** Ewa Jednacz, Lidia Rutkowska-Sak

**Affiliations:** Paediatric Clinic of Rheumatology, Institute of Rheumatology, Spartańska 1, 02-637 Warsaw, Poland

## Abstract

Atherosclerosis is a chronic inflammatory disease of the arteries. Clinical consequences of the atherosclerotic process occur in the adult population, however atherosclerotic process begins in childhood. The classic risk factors for atherosclerosis include obesity, dyslipidaemia, age, gender or family history. In recent years, attention has been drawn to the similarity between atherosclerotic inflammatory processes and inflammatory changes in the course of systemic connective tissue disease, in particular systemic lupus etythematosus (SLE) or rheumatoid arthritis (RA). There is also observed the similarity of the pathogenetic background of development of atherosclerosis and juvenile idiopathic arthritis (JIA). Elevated levels of pro-inflammatory cytokines are observed in the course of juvenile idiopathic arthritis. Also homocysteine concentrations, which may play a significant role in the development of atherosclerotic lesions, are observed higher in patients with JIA. Some studies revealed higher carotid intima-media thickness (IMT) index values in children with JIA. In view of the fact that atherosclerotic process begins as early as in childhood, the introduction of appropriate preventive measures in children is a matter of utmost importance.

## 1. Introduction

Atherosclerosis is a chronic inflammatory disease of the arteries. Clinical consequences of the atherosclerotic process, in the form of ischaemic heart disease, disorders of cerebral circulation, or circulatory disorders of peripheral arteries occur in the adult population; however atherosclerotic changes have their beginning in childhood. The severity of atherosclerosis correlates with the number and intensity of risk factors such as body mass index (BMI), systolic and diastolic arterial blood pressure, total cholesterol, LDL, HDL, triglyceride concentrations, and passive and active cigarette smoking. At present, much significance is attached to the inflammatory aetiology of atherosclerosis, which makes it an inflammatory disease, a vascular wall response to injury. Proinflammatory cytokines, such as IL-1b, IL-6, IL-8, or TNF-*α*, play a significant role in the development and progression of atherosclerotic lesions. Elevation of the concentrations of acute phase proteins, such as CRP, is also a reflection of the inflammatory process. An elevated homocysteine concentration also increases the risk of developing cardiovascular diseases. Awareness of the fact that initiation of the atherosclerotic process takes place very early in life underscores the need for identifying these changes as early as possible despite the absence of clinical symptoms. Special attention must be given to children belonging to the risk group for cardiovascular disease, which includes children with the following medical problems: familial hypercholesterolaemia, diabetes, chronic kidney disease, a past history of neoplastic disease, Kawasaki disease, congenital heart disease, history of a heart transplantation, and chronic inflammatory diseases. Chronic inflammatory diseases include juvenile idiopathic arthritis (JIA); typically, this disease begins before the age of 16 years and has several clinical presentations, of which the most common is the oligoarticular-onset JIA, affecting 1 to 4 joints, and less common subtypes: polyarticular-onset JIA (inflammation of 5 or more joints), systemic JIA and psoriatic arthritis, enthesitis-related arthritis, and forms that do not meet the criteria for the abovementioned subtypes or comprising features of more than one of these subtypes [1].

## 2. The Beginning of the Atherosclerotic Process

The atherosclerotic process begins in childhood, and even in utero. Evidence of this was found in studies by Italian pathologists, headed by Napoli and Palinski [2–4]. They examined 82 aortas obtained from spontaneously aborted foetuses (*n* = 35) and preterm newborns who had died soon after birth (*n* = 47). The mean age of the examined foetuses was 6.2+/− 1.3 months. Some of the mothers of the examined foetuses had a history of hypercholesterolaemia prior to and during the course of their pregnancy, some only during pregnancy, while others had no history of hypercholesterolaemia. Atherosclerotic lesions were present in 60–80% of the examined aortas, irrespective of whether the mothers of these foetuses had normal cholesterol concentrations or hypercholesterolaemia, and the rate of progression of these changes corresponded to the severity of hypercholesterolaemia. 

In the FELIC (Fate of Early Lesions in Children) study, Napoli et al. assessed the aortas of 156 children, aged 1 to 13 years, who had died as a result of trauma or other sudden causes. The children were stratified according to maternal cholesterol status (normocholesterolaemic or hypercholesterolaemic) during pregnancy. All of the children had normal serum cholesterol concentrations. Nonetheless, atherosclerotic lesions in the abdominal aorta and the aortic arch, whose progression advanced with age, were present in all the children; moreover, the rate of progression of these lesions was higher in children of hypercholesterolaemic mothers versus that in children whose mothers were normocholesterolaemic [5].

The Bogalusa Heart Study was an extensive epidemiological study evaluating cardiovascular risk factors in both African Americans and Caucasians, conducted in Louisiana (USA) in the years 1972–2005. In 1998, autopsy reports of 204 individuals who had died from sudden causes were published as part of this study. Fatty streaks were present in all the examined aortas. The presence of fatty streaks in the coronary arteries increased with age and was seen in 50% of children aged 2–15 years and in 85% of individuals in the 26- to 39-year age group. These studies also revealed an association between the extent of atherosclerotic lesions and the presence of risk factors for atherosclerosis (BMI, systolic blood pressure, TG and LDL concentrations, and cigarette smoking) [6]. Furthermore, a 12-year followup of children in the course of the Bogalusa Heart Study revealed that after 12 years approximately 50% of these children still had elevated total cholesterol or LDL concentrations (exceeding the 75th percentile) [7].

The prevalence of atherosclerotic lesions in young individuals was also assessed in the multicentre Pathobiological Determinants of Atherosclerosis in Youth (PDAY) study. The study group consisted of approximately 3,000 individuals who had died aged 15 to 34 years as a result of accidents, suicide, or homicide. Fatty streaks were present in all the adolescents. The degree of progression of atherosclerotic lesions increased with age. Lesion size and the degree of progression were higher in men than in women. The risk factors for developing atherosclerotic lesions were obesity, smoking, and arterial hypertension. Ten percent of the examined young individuals were found to have very advanced atherosclerotic lesions and 80% of them were cigarette smokers [8].

## 3. The Classic Risk Factors for Atherosclerosis

The presented studies, which were based on autopsy results, reveal not only the presence of atherosclerotic lesions, but also a correlation between these changes and the established risk factors for cardiovascular disease: BMI, arterial hypertension, serum lipoprotein concentration, and cigarette smoking. The earliest possible identification of increased-risk groups that should receive particular preventive care becomes of utmost importance. In December 2006, the American Heart Association (AHA) published guidelines endorsed by the American Academy of Pediatrics. Children with the following diseases were identified as being at high risk for cardiovascular disease: familial hypercholesterolaemia, diabetes, chronic kidney disease, a past history of neoplastic disease, Kawasaki disease, congenital heart disease, history of a heart transplantation, and chronic inflammatory diseases. The abovementioned diseases were then divided into 3 groups according to the level of risk. Chronic inflammatory disease was classified as a moderate-risk disorder [9]. March 2010 saw the publication of the Consensus of the Polish Forum for Prevention of Cardiovascular Diseases Task Force regarding prophylactic measures against cardiovascular diseases in children and adolescents, which presented guidelines enabling identification of groups at increased risk and subsequent, appropriate nonpharmacological and pharmacological management [10].

The classic risk factors for atherosclerosis have been well known for years. They include both factors that are amenable to modification, such as obesity or dyslipidaemia, which allow for the introduction of appropriate preventive measures, as well as factors that are not amenable to modification, such as age, gender, or family history, which makes it possible to establish the group at high risk for cardiovascular disease. 

## 4. Atherosclerosis as the Inflammatory Disease

In 1999, Russell Ross presented his ground-breaking hypothesis of an inflammatory aetiology of atherosclerosis [11]. According to this hypothesis, the first stage in the development of atherosclerosis is endothelial dysfunction. The inflammatory reaction is initiated directly by the effect of oxidatively modified LDL on endothelial cells. Lymphocytes and macrophages, which are a source of chemokines, cytokines, growth factors, and proteolytic enzymes, play a major role in the development of atherosclerotic plaques. Proinflammatory cytokines, such as IL-1b, IL-6, IL-8, or TNF-*α*, play a significant role in the development and progression of atherosclerotic lesions. The inflammatory process is also reflected by an increase in the concentration of acute phase proteins, such as C-reactive protein (CRP).

Chronic inflammatory diseases are rheumatic diseases—such as rheumatoid arthritis or juvenile idiopathic arthritis, diseases with multifactorial aetiology and pathogenesis. In recent years, attention has been drawn to the similarity between atherosclerotic inflammatory processes and inflammatory changes in the course of systemic connective tissue diseases, in particular systemic lupus erythematosus or RA. Persistently elevated CRP values in the course of RA increase the risk of death due to cardiovascular disease [12]. CRP determined using high-sensitivity assays appears to be an independent and very robust prognostic factor for cardiovascular events. CRP is not only an indicator for generalized inflammatory reaction, but also a mediator involved in the pathogenesis of atherosclerosis. CRP has been found to correlate with the degree of subclinical atherosclerosis, measured by carotid artery intima-media wall thickness, and the presence of clinically evident cardiovascular disease in adults with rheumatoid arthritis [13, 14].

Cardiovascular disease is the main cause of mortality in patients with RA [15]. Compared with the general population, patients diagnosed with RA have a 60% higher risk of experiencing a first episode of cardiovascular disease [16], and a 3-fold higher risk of myocardial infarction in the case of disease of over 10 years duration [17]. Dyslipidaemia is one of cardiovascular risk factors in RA. It is defined as higher total cholesterol and/or triglycerides and/or lower high-density lipoprotein (HDL) cholesterol levels. The dyslipidaemia in RA is dependent on disease activity. A higher disease activity is associated with lower total cholesterol levels, and even more depressed HDL cholesterol levels [18]. Studies conducted by Lakatos in patients with RA (26 men and 103 women) revealed higher LDL values along with lower HDL and TG concentrations compared to those in the control group (625 men and 749 women) [19].

## 5. Similar Pathogenesis of Atherosclerosis ****and Juvenile Idiopathic Arthritis

At the same time, attention is being drawn to the similarity of the pathogenetic background of development of atherosclerosis and JIA. Children with JIA are seen to have lipid metabolism dysfunction. A study by Urban et al. in 25 children with JIA, with early-phase disease and an absence of clinical signs of obesity, revealed increased concentrations of total cholesterol, LDL, and triglycerides; decreased concentrations of HDL; as well as correlations between homocysteine and total cholesterol concentrations, and homocysteine and LDL [20].

Studies by Gonçalves conducted in a group consisting of 28 children with the polyarticular subtype of JIA revealed decreased HDL concentrations in the serum of 57% of patients with polyarticular JIA and elevated LDL values in 18%; 14% of the children had elevated triglyceride levels while 7% had elevated total cholesterol values. There was no association between reduced HDL concentrations and disease activity, the duration of the disease or the treatment administered [21]. Studies by Tselepis, conducted on a group of 26 children with active JIA, revealed lower plasma total cholesterol and HDL levels and higher plasma triglyceride levels as compared with controls [22]. However, studies conducted by Bakkaloglu, involving a group of 37 children with JIA compared with a group of 18 healthy children did not reveal statistically significant differences in total cholesterol, triglyceride, or HDL concentrations [23].

Homocysteine is a sulphur-containing amino acid produced in the body in the course of methionine conversion, obtained from food. An increase in homocysteine concentration may be caused by a genetically determined deficiency or absence of enzymes involved in the metabolism of homocysteine, a deficiency of coenzymes participating in the conversion of homocysteine, and medications, among them methotrexate, which is the dihydrofolate reductase inhibitor. Methotrexate is the drug most commonly used to treat juvenile idiopathic arthritis. Regarding genetic influence, it is known that a genetic polymorphism—MTHFR 1298 A/C—associated with changes in the levels of homocysteine was also found to influence the development of endothelial dysfunction and increased risk of cardiovascular disease in adults with RA [24]. Postmortem studies by McCully, published in 1969, conducted in children that had died as a result of homocystinuria, gave rise to the hypothesis that an excess of homocysteine may play a significant role in the development of atherosclerotic lesions [25]. Since the late 1960s there have been many studies conducted that confirm this hypothesis. Among others, studies by Gonçalves revealed that homocysteine concentrations are higher in patients with JIA; at the same time, however, no correlation was found between the administration of methotrexate and homocysteine concentrations [26]. Meanwhile, studies by Pietrewicz in children with JIA revealed higher and statistically significant concentrations of homocysteine compared to the control group and higher concentrations of homocysteine in the polyarticular subtype compared with the oligoarticular presentation, particularly among children treated with methotrexate [27]. However, methotrexate use has been associated with a decrease of cardiovascular mortality in adults with rheumatoid arthritis. Therefore, the anti-inflammatory effect mediated by this drug may compensate for the potential ominous effect mediated by the increase of homocysteine levels [28].

Proinflammatory cytokines play a significant role in the development of atherosclerotic lesions [29]. Elevated levels of proinflammatory cytokines are observed in the course of juvenile idiopathic arthritis [30]. Studies by Prahalad revealed that sCD154, IL-1b, IL-5, IL-6, IL-8, IL-13, INF-gamma, sIL-2R, and TNF-alpha concentrations were higher than in the control group [31]. Studies conducted by Yilmaz evaluated IL-1b, IL-6, IL-8, IL-12, and TNF-alpha concentrations in patients with various forms of JIA: systemic, polyarticular, and oligoarticular, during periods of exacerbation as well as in remission. Patients with systemic disease were found to have the highest IL-1b and IL-6 concentrations, both during exacerbations and while in remission. The concentration of these cytokines was higher during periods of exacerbation than that in remission, and cytokine levels during remission were higher than those in the control group. IL-8 and TNF-alpha concentrations in periods of exacerbation and remission were comparable to control group values [32]. The macrophage migration inhibitory factor (MIF) is an immunoregulatory cytokine with proinflammatory properties. It plays a significant role in the pathogenesis of atherosclerosis and the development of advanced atherosclerotic lesions [33]. At the same time, high MIF concentrations are found in the serum and synovial membrane of patients with RA [34]. In a study by Morand, MIF concentrations in the synovial membrane of patients with RA correlated with disease activity, while a reduction in the activity of the inflammatory process was associated with a decrease in MIF levels [35]. However, genetic polymorphisms encompassing the whole IL-6 gene in a large series of patients with RA [36] and a study of the MIF-173 gene polymorphism [37] did not show association with cardiovascular disease in Spanish patients with RA.

The problem of finding noninvasive methods of assessing the risk of developing atherosclerosis in children with risk factors for atherosclerosis, including children with JIA, is answered by carotid intima-media thickness (IMT) assessment via ultrasonographic imaging. A rent meta-analysis confirmed that IMT is increased in adults with RA [38]. This is of particular relevance as an abnormally increased carotid IMT was also found to predict the risk of cardiovascular events in the followup of adults with RA [39]. Studies by Pietrewicz conducted in a group of 40 children with JIA revealed higher IMT index values compared to those in the control group and higher IMT index values in the polyarticular subtype compared to the oligoarticular presentation [27]. Studies conducted by Urban in a group of 63 children (40 with JIA and 23 healthy children) revealed statistically significant differences compared to the control group [40].

Carotid intima-media thickness represents the combined thickness of the intimal and medial layers of carotid artery. The method showing changes in the microcirculation is the capillaroscopy, a simple, safe, and easy technique. It is a good method for detecting microvascular abnormalities in disorders associated with Raynaud's phenomenon. The presence of microangiopathy, ascertained using this technique, appears to be associated with endothelial dysfunction, the earliest form of the atherosclerotic process. However, definitive conclusions regarding the usefulness of capillaroscopic examination requires further studies [41].

## 6. Summary

At present, there are many known risk factors for developing atherosclerosis. Some of them are amenable these may be modified, while some are nonamenable, these cannot be modified; however, they make it possible to identify groups at increased risk for cardiovascular diseases. The list of new factors is constantly growing. In view of the fact that clinical consequences of the atherosclerotic process manifest themselves in the adult population while the atherosclerotic process itself begins as early as in childhood, the introduction of appropriate preventive measures in children, particularly children belonging to the risk group for cardiovascular disease, including children with JIA, is a matter of utmost importance. These measures may halt the development of the atherosclerotic process at a very early stage. However, further studies are required that would enable assessment of the risk of early development of atherosclerosis in children, including children with JIA, particularly since attention is being increasingly drawn to the similar pathogeneses of atherosclerosis and JIA.
